# Different aspects of myocardial intervention and pharmacological treatment

**DOI:** 10.1093/ehjcvp/pvaf092

**Published:** 2026-02-02

**Authors:** Stefan Agewall

**Affiliations:** Institute of Clinical Sciences, Karolinska Institute of Danderyd, Stockholm, Sweden

**Figure:**
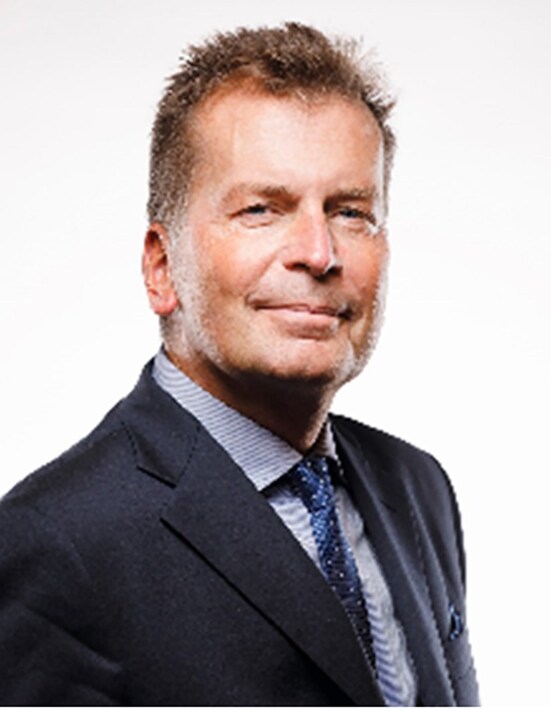


Dear Reader,

This is year 12 of the journal. Time flies. In issue 1, we publish a couple of Pharmapulse reports from recent large studies.

In acute heart failure (AHF) patients, different diuretic strategies are used,^1^ and new ones have been introduced.^2^ Dr Ameri and co-workers from Italy systematically searched phase 3 randomized clinical trials (RCTs) evaluating diuretic regimens in admitted AHF patients within 48 h and irrespective of clinical stabilization. In 25 selected RCTs with 7149 patients, the authors found that continuous Furosemide and sequential nephron blockade (SNB) improve surrogates of response to bolus of furosemide in AHF. Sequential nephron blockade is also connoted by worsening renal function and may induce hypokalaemia.

Dr Yari and co-workers used data from a nationwide Swedish study and examined how well patients continue taking recommended heart medications^3^ after a first heart attack. More than 159 000 patients were followed for up to 12 years after discharge on statins, beta-blockers, aspirin, or Renin–Angiotensin–Aldosteron–System inhibitors. Nearly all patients initially filled their prescriptions. Although many patients stopped treatment at some point, a large proportion later restarted their medication. When these treatment interruptions and restarts were taken into account, most patients were still on therapy long term. About 90% were on treatment at 1 year and roughly 75% at 12 years after the heart attack. The findings suggest that long-term use of preventive heart medications is better than previously thought and that temporary discontinuation is common but often reversible.

In a network meta-analysis, D'Amario *et al*. from Italy evaluated 64 randomized controlled trials including 27 243 STEMI patients undergoing primary PCI with adjunctive intracoronary therapies. No adjunctive pharmacological or mechanical strategy reduced all-cause mortality, recurrent myocardial infarction, or heart-failure hospitalization compared with conventional PCI during ∼8 months’ follow-up. Several treatments (adenosine, verapamil, tirofiban, manual thrombus aspiration, and selected combinations) improved surrogate markers of coronary microvascular obstruction, such as post-PCI thrombolysis in myocardial infarction flow and ST-segment resolution. However, these angiographic and electrocardiographic benefits did not translate into improved clinical outcomes. Safety signals emerged, with intracoronary tirofiban increasing bleeding risk and adenosine increasing atrioventricular block, while nicorandil reduced peri-procedural ventricular arrhythmias. Overall, routine use of intracoronary adjunctive therapies during primary PCI is not supported by current evidence.

Dr Liu and co-workers used a drug-target Mendelian randomization to explore whether genetic proxies of GLP-1 receptor agonist (GLP-1RA) targets are associated with gastrointestinal adverse events, particularly acute pancreatitis.^4,5^ Increased genetically predicted cannabinoid receptor 1 expression in blood was associated with a lower risk of acute pancreatitis, suggesting a potential protective pathway. No robust associations were found for other gastrointestinal outcomes. The authors emphasize that these findings reflect lifelong genetic variation rather than direct pharmacological effects of GLP-1RAs. Overall, the study is hypothesis-generating and highlights possible biological mechanisms warranting further clinical and experimental investigation.

Dr Tzikas and co-workers evaluated the efficacy and safety of antithrombotic strategies following percutaneous left atrial appendage occlusion (LAAO)^6,7^ in patients with non-valvular atrial fibrillation in a network meta-analysis from a total of 52 studies (49 observational and 3 randomized controlled trials) comprising 69 751 patients. The analysis compared vitamin K antagonists, single and dual antiplatelet therapy, and standard- and low-dose direct oral anticoagulants (DOACs). The authors concluded that DOACs provide a superior safety-efficacy profile compared to other antithrombotic strategies following LAAO, significantly reducing the risks of major bleeding, thromboembolic events, and mortality. While low-dose DOACs may offer additional bleeding risk reduction without compromising efficacy, further research is warranted to confirm their role in clinical practice.

## Data Availability

Not applicable.
